# Antioxidant and Antidiabetic Effect of Aqueous Fruit Extract of *Passiflora ligularis* Juss. on Streptozotocin Induced Diabetic Rats

**DOI:** 10.1155/2014/130342

**Published:** 2014-12-21

**Authors:** Palanirajan Anusooriya, Deivasigamani Malarvizhi, Velliyur Kanniappan Gopalakrishnan, Kanakasabapathi Devaki

**Affiliations:** ^1^Department of Biochemistry, Karpagam University, Coimbatore, Tamil Nadu 641 021, India; ^2^Department of Bioinformatics, Karpagam University, Coimbatore, Tamil Nadu 641 021, India

## Abstract

Diabetes mellitus is the most common endocrine disorder that impairs glucose homeostasis resulting in severe diabetic complications including retinopathy, angiopathy, nephropathy, and neuropathy causing neurological disorders due to perturbation in utilization of glucose. Hypoglycemic activity was detected in aqueous extract of* Passiflora ligularis*, a traditionally used medicinal plant, using streptozotocin (STZ, 30 mg/kg body weight) induced diabetic rat model. Oral administration of aqueous extract of* Passiflora ligularis* to diabetic rats for 30 days resulted in a decrease in blood glucose. The diabetic rats had decreased levels of serum total protein, albumin, globulin, and albumin/globulin ratio as compared to control rats. In addition, the activities of hepatic and renal markers were significantly elevated in diabetic rats as compared to control rats. Treatment with aqueous fruit extract of* P. ligularis* and glibenclamide reversed these parameters to near normal. Extract at a dose of 400 mg/kg given orally for 30 days showed significant elevation in enzymatic (SOD, catalase, and Gpx) and nonenzymatic antioxidants (vitamin C, vitamin E, and reduced glutathione). Plant extract treated groups showed significant decrease in lipid peroxidation (LPO). Aqueous extract of* Passiflora ligularis* fruit can decrease the blood glucose and reduce the oxidative stress by removing free radicals in diabetes.

## 1. Introduction 

Diabetes mellitus is a metabolic disorder in which a combination of hereditary and environmental results in abnormally high blood sugar levels (hyperglycemia). The abnormal high blood sugar level is due to defects in either insulin secretion or insulin action in the body [1]. Diabetes mellitus is characterized by hyperglycemia, lipoprotein abnormalities, raised basal metabolic rate, defect in enzymes, and high oxidative stress which induced damage to pancreatic beta cells. It is the most common endocrine disorder that impairs glucose homeostasis resulting in severe diabetic complications including retinopathy, angiopathy, nephropathy, and neuropathy and causing neurological disorders due to perturbation in utilization of glucose [2].

Currently, in the United States, up to 1 in 3 new cases of diabetes mellitus diagnosed in youth younger than 18 years is T2DM, with a disproportionate representation in ethnic minorities, occurring most commonly among youth between 10 and 19 years of age. This trend is not limited to the United States but is occurring internationally; it is projected that by the year 2030, an estimated 366 million people worldwide will have diabetes mellitus [3]. The number of individuals with type 2 diabetes mellitus (T2DM) is increasing with a rate of three new cases every ten seconds [4], and it is being diagnosed at younger age. Multiple risk factors behind the disease include chronic stress and depression, environmental pollutants and poisons, obesity and overnutrition, and sedentary life style [5]. India having the highest number of diabetic patients in the world, the sugar disease is posing an enormous health problem in the country. Calling India the diabetes capital of the world, the International Journal of Diabetes in Developing Countries says that there is alarming rise in diabetes in India. An estimated 3.4 million deaths occur due to consequences of high blood sugar. WHO also estimates that 80 percent of diabetes deaths occur in low- and middle-income countries and projects that such deaths will double between 2005 and 2030 [6].

Species of* Passiflora *are commonly found all over the world. Studies have revealed its use in anti-inflammatory, antimicrobial, antioxidant, and anti tumor process. Various types of preparations, extracts, and individual compounds derived from this species have been found to possess a broad spectrum of pharmacological effects on several organs such as the brain, blood, and cardiovascular and nervous systems as well as on different biochemical processes and physiological functions including proteosynthesis, work capacity, reproduction, and sexual function. Studies are needed to examine the potential use of species of* Passiflora *extract in the prevention of pathologies, such as cardiac ischemia, renal ischemia, and neurodegenerative diseases and diabetes, where oxidative stress damage to protein seems to play a major role.

Medicinally, the fruit has been used in Amazonia as a preventative for yellow fever, gallstones, rabies, and ulcers. That region also prescribes a leaf decoction for preventing malaria and other fevers and for easing stomach upsets. Passion fruit is proved to have analgesic (pain-reliever), antianxiety, anti-inflammatory, antispasmodic, cough suppressant, aphrodisiac, central nervous system depressant, diuretic, hypotensive (lowers blood pressure), and sedative activities. Besides, it is traditionally reported to possess anticonvulsant, antidepressant, astringent, cardiotonic (tones, balances, and strengthens the heart), disinfectant, nervine (balances/calms nerves), neurasthenic (reduces nerve pain), tranquilizer, and vermifuge (expels worms) activities. It may have promising and powerful effects on neurological disorders and chronic diseases such as heart disease and cancer [7]. So far, no study has been carried out to reveal the antihyperglycemic effect of* P. ligularis*. This study was undertaken to find out antioxidant and antidiabetic potential in aqueous fruit extract of* P. ligularis.*

## 2. Materials and Methods

### 2.1. Collection and Extraction of Plant Material

Fresh fruits of* Passiflora ligularis* were collected from Munnar, Kerala, and authenticated by Dr. G. V. S. Murthy Botanical survey of India, Tamil Nadu Agricultural University, Coimbatore (Voucher number BSI/SRC/5/23/2012/Tech/495). The fruits were washed thoroughly with water and the peel was removed; then the pulp was collected. The fruit pulp was then grounded in an electric mixer. The fruit juice was collected and filtered. The aqueous extract was concentrated using a rotary evaporator at room temperature for 3 days to obtain a dark brown pigment and weighed. Then the aqueous extract was stored in an air tight container for future use.

### 2.2. Preliminary Phytochemical Screening

Phytochemical screening was done for analyzing the presence of secondary metabolites that are responsible for curing ailments [8, 9].

### 2.3. Experimental Animals

Adult male albino rats weighing about 150–200 g were obtained from the animal house of Karpagam University, Coimbatore, and used for the study. Rats were housed at constant temperature of 22 ± 5°C with a 12-hour light, 12-hour dark cycle, and fed on pellets with free access to tap water. All the experiments were carried out according to the guidelines recommended by the Committee for the Purpose of Control and Supervision of Experiments on Animals (CPCSEA), Government of India.

### 2.4. Acute Toxicity

The Wistar albino rats weighing between 150 and 180 g were used for the study. 250, 500, 1000, and 2000 mg/kg of the aqueous extract of* Passiflora ligularis* were administered orally to four groups of five animals each. Another group of five rats served as control and this received 1 ml of physiological saline. They were all placed under observation for 24 hours for the signs of lethality, toxic symptoms, behavioral changes, or deaths.

### 2.5. Induction of Diabetes

Diabetes was induced by a single intraperitoneal injection of streptozotocin (30 mg/kg) in water. Hyperglycemia was confirmed after 72 hrs by the elevated blood glucose and the behavioral changes (excess thirst and frequent urination). The rats with blood glucose level more than 240 mg/dL were used for the study.

## 3. Glucose Tolerance Test** [10]**

### 3.1. Experimental Design for GTT of Diabetic Rats

The glucose tolerance test was studied in the aqueous extract of* Passiflora ligularis* on diabetic rats. The animals were divided into 5 groups (*n* = 3) as follows:

 Group 1: Control rats;
 Group 2: Diabetic control rats;
 Group 3: Rats treated with 200 mg/kg of aqueous extract of fruit of* P. ligularis*;
 Group 4: Rats treated with 400 mg/kg of aqueous extract of fruit of* P. ligularis*;
 Group 5: Rats treated with 600 mg/kg of aqueous extract of fruit of* P. ligularis*.

The animals were fasted overnight with free access to water. Fasting blood sample was drawn from the tail and blood glucose level was measured by using heamoglucostrips in glucometer (Life scan, Johnson and Johnson Ltd.) and it was also confirmed by O-Toluidine method. For GTT, control and diabetic control rats were given water only. Group 3, 4, 5 rats were treated with their corresponding concentration 200, 400, and 600 mg/kg of aqueous fruit extract of* P. ligularis*, respectively. The blood glucose level was checked after 30 minutes. Then all the rats were loaded with 3 g/kg glucose solution. Three more blood samples were collected at 60, 120, and 180 minutes after the glucose load.

Glycemic index was calculated by using formula

(1) $$\begin{array}{c} \text{Glycemic}\;\text{index}=\frac{\text{Initial}-\text{Final}}{\text{Initial}}\times 100. \end{array}$$

### 3.2. Antidiabetic Study

The animals were divided into five groups with four rats in each group. Group 1 served as control, normal healthy rats given normal pelleted diet and 1.0 ml citrate buffer as vehicle, group 2 rats were induced with diabetes by a single intraperitoneal injection of 30 mg/kg bw of streptozotocin and kept without any treatment for 30 days, group 3 rats were induced with diabetes as mentioned in group 2 and treated with glibenclamide (1.25 mg/kg bw) orally through oral intragastric tube, for a period of 30 days, group 4 rats were induced with diabetes as mentioned in group 2 and treated with* Passiflora ligularis* (400 mg/kg bw) orally for a period of 30 days, group 5 rats were treated with* Passiflora ligularis* alone (400 mg/kg bw) orally for a period of 30 days, and the fruit aqueous extract (400 mg/kg) was given orally through intragastric tube for 30 days.

The extract was administered orally by intragastric tubes for the study period of 30 days and then they were sacrificed under mild chloroform anesthesia. The blood and the serum separated from blood were used for biochemical studies. The organs liver, kidney, and pancreas were collected in saline and used for antioxidant analysis.

### 3.3. Biochemical Analysis

Serum glucose was estimated by kit method [11]. Hemoglobin and glycosylated hemoglobin was estimated by Drabkin's method [12]. Serum cholesterol and HDL was estimated by one step method [13] using diagnostic reagent kit manufactured by Span Diagnostics Ltd. Triglycerides were estimated by GPO-PAP, end point assay [14] using diagnostic reagent kit manufactured by Span Diagnostics Ltd. The activities of serum aspartate aminotransferase (AST) and alanine aminotransferase (ALT) were estimated by using commercially available kits [15]. The activities of serum alkaline phosphatase were also estimated [16]. Total protein and albumin in the serum were estimated by Biuret method [17]. Urea in the serum was estimated by using the diagnostic kit based on the DAM method [18]; creatinine in serum was estimated by using the diagnostic kit based on the alkaline picrate method [19]. Bilirubin in serum was estimated by using the Span Diagnostic kit [20].

### 3.4. Antioxidant Analysis in Tissues

In the present study, antioxidant activity of aqueous fruit extract of* Passiflora ligularis* was analyzed in different organs, namely, liver, kidney, and pancreas. After 30 days the rats were sacrificed under mild chloroform anesthesia and the organs were collected in saline liver which was used for the estimation of glycogen [21]. All the tissues were used for estimation of protein [22], enzymic antioxidants such as superoxide dismutase [23], catalase [24], GPx [25], and nonenzymatic antioxidants such as vitamin C [26], vitamin E [27], and reduced glutathione [28]. The lipid peroxidation [29] was also estimated.

### 3.5. Statistical Analysis

The results obtained were expressed as mean ± standard deviation (SD). The statistical comparison among the groups was performed with one-way analysis of variance followed by Duncan's Multiple Range Test (DMRT) using SPSS version 10 (SPSS, Chicago, IL). The limit of statistical significance was set at *P* < 0.05.

## 4. Results and Discussion

Phytochemicals play an important role in plant defense against prey, microorganism, and stress as well as interspecies protections. These plant components have been used as drugs for millennia. Hence, phytochemical screening serves as the initial step in predicting the types of potential active compounds from plants [30].  Table 1 shows the phytochemicals present in aqueous fruit extract of* P. ligularis.*

Phytochemical screening of* P. ligularis* revealed the presence of various phytochemicals (Table 1). In particular the aqueous extract of* Passiflora ligularis* revealed the presence of alkaloids, tannins, phenolic compounds, flavonoids, steroids, cardiac glycosides, terpenoids, and carbohydrates.

In acute toxicity study, the experimental rats had slept several hours, after administration of* Passiflora ligularis *extract to the Wistar albino rats when compared to normal control rats. But there were no gross behavioral changes or morphological changes like respiratory distress, hair loss, restlessness, convulsions, laxative, coma, weight loss, urination, itching, and so forth. There was no lethality and no toxic reaction was found at any of the doses selected till the end of the treatment. This indicates the safety nature of* Passiflora ligularis *extract on Wistar albino rats.

**Figure 1:**
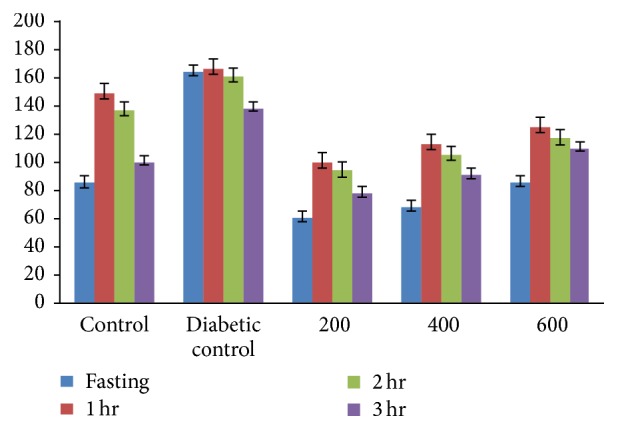
Oral glucose tolerance test of aqueous extract of* Passiflora ligularis* in experimental rats.

**Figure 2:**
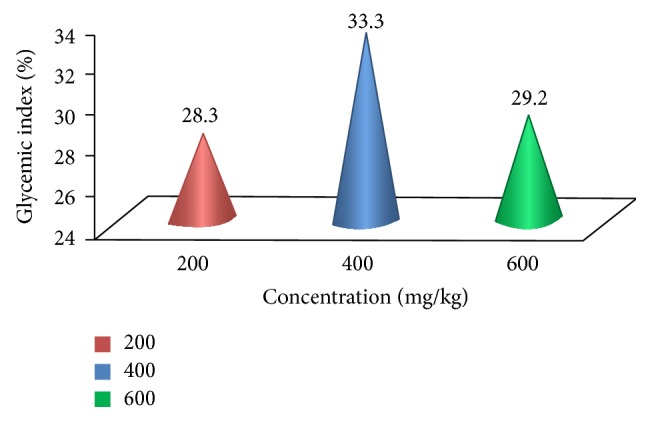
The glycemic index of aqueous extract of* Passiflora ligularis.*

Figure 1 shows the blood glucose levels in GTT of control and experimental groups of rats after oral administration of glucose. The aqueous fruit extract of* Passiflora ligularis *was administered orally (200, 400, and 600 mg/kg, for 15 days) to experimental animals. In diabetic rats, the peak increase in blood glucose concentration was observed after 60 min and it remained high over 120 min.* Passiflora ligularis *treated diabetic rats showed significant decrease in blood glucose concentration at 60 min and at 120 min interval and the glycemic index was found to be 28.3%, 33.3%, 29.2%, respectively (Figure 2). Among the various doses (200, 400, and 600 mg/kg) of* P. ligularis *on OGTT in normal and diabetic rats, 400 mg/kg brought an effective hypoglycemic effect when compared to other doses. This effect may occur due to reduction in intestinal glucose absorption or induction of glycogenic process along with reduction in glycogenolysis and glyconeogenesis. Therefore, this effective dosage, 400 mg/kg of aqueous fruit extract of* P. ligularis*, was used for further antidiabetic studies in Wistar albino rats. The observed diabetic untreated rats were increased in serum glucose in due to the effect of STZ which cause tissue damage in pancreas that destroy *β* cells and result in insulin deficiency. Insulin deficiency ultimately causes increased blood glucose [31].

In diabetic control rats, there is significant elevation of glucose. Streptozotocin causes selective destruction of cells of islets of pancreas and brings an increase in blood glucose levels [32]. It is evident from the present investigation that administration at the dose of 30 mg/kg body weight causes significant diabetogenic response in albino rats. From these results given in Table 2, that reduction in blood glucose levels brought by aqueous extract of* P. ligularis* was quite comparable with reduction brought about by glibenclamide. A significant elevation in hemoglobin and increase in glycosylated hemoglobin noticed in diabetic rats were normalized to near normal with the administration of aqueous fruit extract of* P. ligularis *and glibenclamide. Reduction in hemoglobin in diabetic rats is due to the interaction of excess glucose with hemoglobin to form glycosylated hemoglobin (Table 2). Glycosylated hemoglobin (HbA1c) was almost doubled in STZ rats and it decreased significantly when treated with* P. ligularis* and maintains the hemoglobin and glycosylated hemoglobin in their normal range. This proves the role of* P. ligularis* in controlling the blood glucose.

A significant elevation in serum lipids was observed in diabetic rats when compared with control rats (Table 2). In case of insulin deficiency as in diabetes mellitus, lipolysis is not inhibited and therefore this leads to hyperlipidemia. On oral administration of* Passiflora ligularis* fruit extract to diabetic rats for 30 days significantly reversed these values to near normal. This may be due to the increase in insulin secretion by* Passiflora ligularis* which decreases the total cholesterol and total triglycerides and increases HDL level.

Table 3 shows the level of hepatic and renal markers; the levels of urea, creatinine, and bilirubin were significantly increased in diabetic group and treatment with* Passiflora ligularis* extract for 30 days significantly reversed these values to near normal. Similar effect was observed in glibenclamide treated group.* Passiflora ligularis* extract alone treated rats showed similar effect to that of control rats. The AST and ALP are considered sensitive indicator of liver injury [33]. Rise in serum level of AST and ALP have been attributed to the damaged structural integrity of the liver. The significant decrease in liver enzymes, namely, AST and ALP levels, was noticed after oral administration of aqueous extract of* P. ligularis* as compared to diabetic animals. It implies the normal functioning and protective effect of* P. ligularis *liver and supports hepatoprotective nature of* P. ligularis*.

The results from Table 3 show that the serum total protein level in diabetic control rats was significantly reduced. Increase in serum protein, that is, the ratio of albumin and globulin in diabetic rats treated with aqueous extract of* P. ligularis *and standard drug, was observed. Liver damage is most common in diabetes mellitus. Administration of* Passiflora ligularis* fruit extract decreased the level of liver markers in diabetic treated rats. This shows the hepatoprotective effect of* P. ligularis.*

STZ causes damage to liver, kidney, and pancreas as well as the hyperglycemia related changes which may persist in the tissues. The changes on protein levels in tissues such as liver, kidney, and pancreas of the experimental animals are given in Table 4. The level of protein in liver and kidney was decreased in diabetic group on comparison with control group. On treatment with aqueous fruit extract of* Passiflora ligularis *and standard drug glibenclamide to diabetic rats for 30 days the values were significantly increased to near normal. Aqueous extract of* Passiflora ligularis *alone treated group did not show any adverse changes. The reduction of the level of total proteins in induced rats was attributed to localized damage in the endoplasmic reticulum which results in the loss of P_450_ leading to its functional failure with a decrease in protein synthesis. The rise in protein levels in the treated groups suggests the stabilization of endoplasmic reticulum leading to protein synthesis [34]. Administration of* Passiflora ligularis* may enhance the protein synthesis by stabilizing the endoplasmic reticulum.

**Figure 3:**
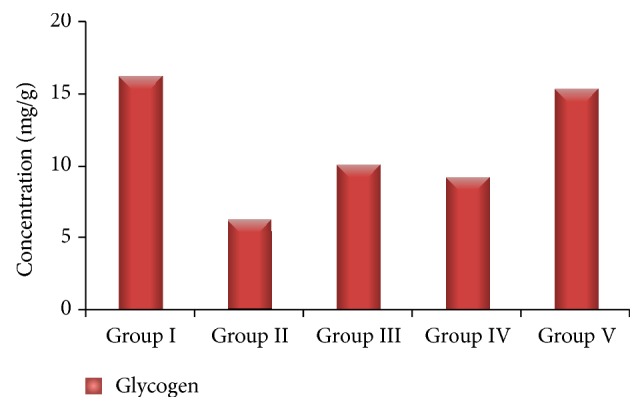
Changes on liver glycogen of control and experimental rats.

In this study, the liver glycogen level was decreased significantly in Group II diabetic rats, compared to glibenclamide as standard and* Passiflora ligularis *treated groups. Liver glycogen content was significantly reduced in STZ induced diabetic rats (Figure 3). Glycogen is the primary intracellular storage form of glucose and its levels in various tissues are a direct reflection of insulin activity as insulin promotes intracellular glycogen deposition by stimulating glycogen synthase and inhibiting glycogen phosphorylase [35]. The significant increase of liver glycogen level in the extract-treated diabetic groups may be due to reactivation of the glycogen synthase system. The experimental results indicate that the aqueous fruit extract of* Passiflora ligularis *has considerable antidiabetic activity and is capable of maintaining the liver glycogen level.

Table 5 demonstrates the results of the antioxidants enzymes levels of SOD, catalase, and GPX in experimental rats. These enzymatic antioxidants are significantly decreased in different organs (liver, kidney, and pancreas) due to the inadequacy of the antioxidant defences in combating ROS mediated damage and when they are treated with aqueous fruit extract of* Passiflora ligularis* the activity of these enzymes was increased and may help to control the free radicals when compared to diabetic rats and the effect produced by aqueous fruit extract of* Passiflora ligularis* was comparable with that of standard drug glibenclamide. Implication of oxidative stress in the pathogenesis of diabetes is suggested, not only by oxygen free-radical generation, but also due to nonenzymatic protein glycosylation, autooxidation of glucose, impaired glutathione metabolism, alteration in antioxidant enzymes, lipid peroxides formation, and decreased ascorbic acid levels. In addition to GSH, there are other defense mechanisms against free radicals like the enzymes superoxide dismutase (SOD), reduced glutathione (GSH), and catalase (CAT) whose activities contribute to eliminate superoxide, hydrogen peroxide, and hydroxyl radicals [36]. The decreased activities of CAT and SOD in diabetic rats may be a response to increased production of H_2_O_2_ and O_2_ by the autoxidation of glucose. These enzymes play an important role in maintaining physiological levels of oxygen and hydrogen peroxide by hastening the dismutation of oxygen radicals and eliminating organic peroxides and hydroperoxides generated from inadvertent exposure to STZ [37]. The observed increases in the antioxidant enzymes in diabetic treated rats are due to the presence of secondary metabolites in the aqueous fruit extract of* Passiflora ligularis. *The aqueous fruit extract* Passiflora ligularis *is rich in flavonoid content which provide to have good antioxidant potential and it is able to reverse the changes in diabetic control rats.

Table 6 indicates a significant reduction in the nonenzymatic antioxidants like glutathione (GSH) vitamins C and E in diabetic rats when compared with control rats. The levels of these antioxidants were significantly increased in different organs (liver, kidney, and pancreas) of diabetic rats by treating with aqueous fruit extract of* Passiflora ligularis. *GSH has a multifaceted role in antioxidant defense. It is a direct scavenger of free radicals as well as a cosubstrate for peroxide detoxification by glutathione peroxidases. Oxidative stress in diabetes decreased the level of GSH in different organs of rat when compared to control. Oral administration of aqueous fruit extract of* Passiflora ligularis* for 30 days showed significant elevation in all the nonenzymatic antioxidants values and reached near normal values. This indicates that administration of aqueous fruit extract of* Passiflora ligularis* can reduce the oxidative stress leading to less degradation of GSH due to less production of ROS in diabetic stage. Significant elevation of GSH level was reported in the* P. corymbosa *(Rottl.) root extract-treated diabetic rats [38].

Table 7 explains a significant reduction in the lipid peroxidation in liver, kidney, and pancreas of control and experimental animals. In liver, kidney, and pancreas tissues of diabetic rats, lipid peroxidation (LPO) levels were elevated significantly as compared to that of control rats. Hydroxyl radicals are the major active species that cause lipid oxidation and significant biological damage [39]. The ability of the* Passiflora ligularis* extracts to quench hydroxyl radicals seems to be directly related to inhibiting the process of lipid peroxidation. After oral administration of* Passiflora ligularis *extract for 30 days the elevated values restore back to near normal level. Both of the treated groups showed significant decrease in lipid peroxidation, suggesting its role in protection against lipid peroxidation.

## 5. Conclusion

In conclusion, the result of this study shows that oral administration of the aqueous extract of* P. ligularis* reduces blood glucose, serum lipids which could be due to improvement in insulin secretion by recovery of pancreatic *β* cells.* P. ligularis* possesses antioxidant potential which may be used for therapeutic purposes mainly in the prevention of oxidative damage that occurs during diabetes. Presence of alkaloids and flavonoids of* P. ligularis* has also been found to be beneficial in controlling diabetes and many other diseases as evident from this study. Therefore, it is concluded that the aqueous extract of* P. ligularis* possesses antidiabetic activity and it may prove to be effective for the management of diabetes.

## Figures and Tables

**Table 1:** Phytochemical screening aqueous fruit extract of *Passiflora ligularis*.

Extracts	AL	SA	TP	FL	ST	CG	OF	TN	AP	CHO
Aqueous	++	++	+	++	++	++	++	+	+	+

AL: alkaloids; CHO: carbohydrates; ST: steroids; CG: cardioglycosides; FL: flavanoids; SA: saponins; TP: tannin and phenolic compounds; OF: oils and fats; AP: amino acids and proteins; TN: terpenoids.

“**+**”: present; “−” absent.

**Table 2:** Effect of *P.ligularis* on glucose, hemoglobin, glycosylated hemoglobin, and lipid profile in serum of control and experimental rats.

Parameters	Glucose (mg/dL)	Hemoglobin (mg/dL)	Glycosylated hemoglobin (%)	Total cholesterol (mg/dL)	Triglycerides (mg/dL)	HDL (mg/dL)
Control (Group I)	96.1 ± 0.28^a^	13.3 ± 0.43^a^	6.5 ± 0.12^a^	111.6 ± 5.77^ a^	77.4 ± 0.40^a^	21.3 ± 0.28^a^
Diabetic control (Group II)	221.6 ± 2.28^b^	6.8 ± 0.05^b^	11.2 ± 0.01^b^	228.3 ± 2.88^b^	188.5 ± 0.46^b^	15.2 ± 0.21^b^
Diabetes + glibenclamide (Group III)	111.6 ± 0.57^c^	13.3 ± 0.04^a^	5.4 ± 0.04^c^	115.6 ± 1.15^a^	101.6 ± 2.88^c^	23.0 ± 0.04^c^
Diabetes + *P. ligularis *extract (Group IV)	116.1 ± 0.28^d^	12.7 ± 0.12^c^	6.3 ± 0.05^d^	131.6 ± 2.88^c^	88.8 ± 0.11^d^	25.1 ± 0.28^d^
*P. ligularis *extract treated alone (Group V)	95.1 ± 0.28^a^	13.4 ± 0.01^a^	6.6 ± 0.02^a^	101.6 ± 2.88^d^	78.8 ± 0.28^a^	21.3 ± 0.28^a^

Values are expressed as mean ± SD for four animals in each group.

Values not sharing common superscript letters (a–d) differ significantly at *P* < 0.05 (DMRT).

**Table 3:** Effect of *P. ligularis* on kidney and liver markers, total protein, albumin, and globulin in serum of control and experimental rats.

Parameters	Urea (mg/dL)	Creatinine (mg/dL)	Bilirubin (mg/dL)	SGOT (IU/L)	SGPT (IU/L)	Total protein (mg/dL)	Albumin (mg/dL)	Globulin (mg/dL)
Control (Group I)	27.9 ± 0.01^a^	0.5 ± 0.06^a^	1.10 ± 0.01^a^	33.3 ± 0.28^a^	27.4 ± 0.40^a^	8.2 ± 0.23^a^	5.5 ± 0.11^a^	2.4 ± 0.67^a^
Diabetic control ** ** (Group II)	30.4 ± 0.46^b^	0.7 ± 0.04^b^	1.33 ± 0.02^b^	56.1 ± 0.98^b^	59.0 ± 0.80^b^	4.6 ± 0.02^b^	3.3 ± 0.02^b^	0.6 ± 0.01^b^
Diabetes + glibenclamide ** **(Group III)	25.4 ± 0.34^c^	0.5 ± 0.01^a^	1.18 ± 0.01^c^	38.4 ± 0.40^c^	33.3 ± 0.23^c^	7.7 ± 0.03^c^	4.8 ± 0.26^c^	2.9 ± 0.05^c^
Diabetes + *P. ligularis *extract ** **(Group IV)	23.2 ± 0.35^d^	0.4 ± 0.01^c^	1.15 ± 0.01^d^	43.3 ± 0.28^d^	41.0 ± 0.86^d^	7.2 ± 0.08^d^	4.7 ± 0.19^c^	2.3 ± 0.06^a^
*P. ligularis *extract treated alone ** **(Group V)	27.6 ± 0.34^a^	0.6 ± 0.01^d^	1.09 ± 0.01^a^	35.1 ± 0.28^e^	29.3 ± 0.28^e^	8.6 ± 0.05^e^	5.4 ± 0.10^a^	3.1 ± 0.01^d^

Values are expressed as mean ± SD for four animals in each group.

Values not sharing common superscript letters (a–d) differ significantly at *P* < 0.05 (DMRT).

**Table 4:** Changes on protein levels in liver, kidney, and pancreas of control and experimental animals.

Particulars	Protein (mg/g)		
	Liver	Kidney	Pancreas
Control (Group I)	1.42 ± 0.07^a^	1.06 ± 0.03^a^	0.93 ± 0.03^a^
Diabetic control (Group II)	0.87 ± 0.01^b^	0.57 ± 0.06^b^	0.50 ± 0.12^b^
Diabetic + glibenclamide (Group III)	1.26 ± 0.04^c^	0.88 ± 0.06^c^	0.80 ± 0.09^c^
Diabetic + *Passiflora ligularis* Group IV	1.34 ± 0.025^cd^	1.01 ± 0.06^ad^	0.91 ± 0.03^a^
*Passiflora ligularis* alone treated (Group V)	1.48 ± 0.16^ad^	1.08 ± 0.06^a^	1.07 ± 0.17^d^

Values are expressed as mean ± SD for four animals in each group.

Values not sharing common superscript letters (a–d) differ significantly at *P* < 0.05 (DMRT).

**Table 5:** Activity of aqueous fruit extract of *Passiflora ligularis* on the antioxidant enzymes in the liver, kidney, and pancreas of control and experimental rats.

Particulars	Superoxide dismutase (enzyme required for 50% inhibition of NBT reduction/min/mg protein)			Catalase (*µ*moles of H_2_O_2_utilized/min/mg/protein)			Glutathione peroxidase (*µ*moles of GSH utilized/min/mg/protein)		
	Liver	Kidney	Pancreas	Liver	Kidney	Pancreas	Liver	Kidney	Pancreas
Control (Group I)	6.71 ± 0.01^a^	2.13 ± 0.03^a^	1.13 ± 0.01^a^	2.04 ± 0.10^a^	2.04 ± 0.10^a^	2.2 ± 0.13^a^	1.04 ± 0.25^a^	1.36 ± 0.3^a^	1.77 ± 0.06^a^
Diabetic control (Group II)	3.93 ± 0.02^b^	0.94 ± 0.03^b^	0.63 ± 0.01^b^	0.8 ± 0.05^b^	0.8 ± 0.05^b^	0.93 ± 0.13^b^	0.52 ± 0.17^b^	0.72 ± 0.2^b^	1.15 ± 0.08^b^
Diabetic+ glibenclamide (Group III)	6.4 ± 0.2^c^	1.93 ± 0.02^c^	0.93 ± 0.02^c^	1.1 ± 0.31^c^	1.18 ± 0.31^c^	1.27 ± 0.51^c^	0.93 ± 0.18^c^	0.78 ± 0.09^b^	1.45 ± 0.2^c^
Diabetic + *Passiflora ligularis* (Group IV)	5.91 ± 0.01^d^	1.76 ± 0.02^d^	0.88 ± 0.01^d^	1.68 ± 0.11^d^	1.68 ± 0.11^d^	1.84 ± 0.41^d^	0.84 ± 0.05^d^	1.12 ± 0.14^c^	1.6 ± 0.06^d^
*Passiflora ligularis* alone treated (Group V)	6.56 ± 0.03^ca^	1.97 ± 0.01^c^	1.16 ± 0.03^a^	1.97 ± 0.13^a^	1.97 ± 0.13^ad^	2.16 ± 0.16^a^	1.04 ± 0.2^a^	1.53 ± 0.19^d^	1.72 ± 0.08^a^

Values are expressed as mean ± SD for four animals in each group.

Values not sharing common superscript letters (a–d) differ significantly at *P* < 0.05 (DMRT).

**Table 6:** Effect of aqueous fruit extract of *Passiflora ligularis* on the nonenzymatic antioxidant in the liver, kidney, and pancreas of control and experimental rats.

Particulars	Vitamin E (*µ*g/mg protein)			Vitamin C (*µ*g/mg protein)			Reduced glutathione (*µ*g/mg protein)		
	Liver	Kidney	Pancreas	Liver	Kidney	Pancreas	Liver	Kidney	Pancreas
Control (Group I)	1.56 ± 0.05^a^	3.3 ± 0.33^a^	3.24 ± 0.16^a^	0.34 ± 0.02^a^	0.33 ± 0.014^a^	0.26 ± 0.025^a^	17.4 ± 0.4^a^	11.89 ± 0.46^a^	11.14 ± 0.52^a^
Diabetic control (Group II)	0.84 ± 0.08^b^	2.26 ± 0.25^b^	1.23 ± 0.16^b^	0.27 ± 0.02^b^	0.25 ± 0.013^b^	0.21 ± 0.02^b^	6.76 ± 0.93^b^	4.13 ± 0.69^b^	5.94 ± 0.46^b^
Diabetic + glibenclamide (Group III)	1.42 ± 0.19^ca^	3.47 ± 0.11^a^	2.58 ± 0.23^c^	0.30 ± 0.01^b^	0.28 ± 0.01^c^	0.25 ± 0.01^a^	11.08 ± 0.46^c^	7.11 ± 0.32^c^	7.13 ± 0.28^c^
Diabetic + *Passiflora ligularis* (Group IV)	1.3 ± 0.5^ca^	3.38 ± 0.19^a^	3.37 ± 0.37^a^	0.32 ± 0.01^a^	0.32 ± 0.025^a^	0.26 ± 0.026^a^	13.11 ± 0.61^d^	8.92 ± 0.81^d^	8.76 ± 0.58^d^
*Passiflora ligularis* alone treated (Group V)	1.4 ± 0.4^ca^	3.15 ± 0.47^a^	3.37 ± 0.22^a^	0.33 ± 0.02^a^	0.34 ± 0.03^a^	0.27 ± 0.014^a^	17.52 ± 0.42^a^	11.62 ± 0.46^a^	10.87 ± 0.42^a^

Values are expressed as mean ± SD for six animals in each group.

Values not sharing common superscript letters (a–d) differ significantly at *P* < 0.05 (DMRT).

**Table 7:** Changes on lipid peroxidation in liver, kidney, and pancreas of control and experimental rats.

Particulars	Lipid peroxidation^*^		
	Liver	Kidney	Pancreas
Control (Group I)	14.9 ± 0.26^a^	9.8 ± 0.2^a^	9.07 ± 0.14^a^
Diabetic control (Group II)	31.1 ± 0.32^b^	16.2 ± 0.18^b^	12.6 ± 0.34^b^
Diabetic + glibenclamide (Group III)	18.8 ± 0.23^c^	12.4 ± 0.53^c^	10.19 ± 0.11^c^
Diabetic + *Passiflora ligularis* (Group IV)	16.9 ± 0.18^d^	13.5 ± 0.35^d^	9.69 ± 0.13^d^
*Passiflora ligularis* alone treated (Group V)	15.4 ± 0.16^a^	9.5 ± 0.21^a^	9.10 ± 0.49^a^

Values are expressed as mean ± SD for six animals in each group.

Values not sharing common superscript letters (a–d) differ significantly at *P* < 0.05 (DMRT).

^*^ *n* moles of MDA formed/min/mg of protein.
